# The intrinsic reason why LANSS, DN4, and PainDETECT questionnaires cannot distinguish neuropathic pain from nociplastic pain

**DOI:** 10.3389/fpain.2025.1658126

**Published:** 2025-08-21

**Authors:** Jean-Pascal Lefaucheur

**Affiliations:** ^1^ UR4391 (ENT Team), Faculty of Health, Paris Est Créteil University, Créteil, France; ^2^ Department of Clinical Neurophysiology, Henri Mondor University Hospital, AP-HP, Créteil, France

**Keywords:** diagnosis, neuropathic pain, nociceptive pain, nociplastic pain, central sensitization

In 1994, the Task Force on Taxonomy of the International Association for the Study of Pain (IASP) published the first classification of chronic pain (1), introducing the concept of “neuropathic pain”, defined as “pain initiated or caused by a primary lesion or dysfunction in the nervous system”. From this date, two mechanisms of chronic pain have been considered, either neuropathic or non-neuropathic (nociceptive) pain. In 2011, the IASP Terminology Working Group (2) updated the definitions of “neuropathic pain”, as “pain caused by a lesion or disease of the somatosensory nervous system”, and “nociceptive pain”, as “pain that arises from actual or threatened damage to non-neural tissue and is due to the activation of nociceptors” occurring with a normally functioning somatosensory nervous system. In 2017, based on a publication by the Terminology Task Force of the IASP (3), the IASP Council adopted the proposal for a third mechanistic descriptor of chronic pain, entitled “nociplastic pain”, defined as “pain that arises from altered nociception despite no clear evidence of actual or threatened tissue damage causing the activation of peripheral nociceptors or evidence for disease or lesion of the somatosensory system causing the pain” (4).

The diagnosis of chronic neuropathic pain, which is now a defined clinical entity (5), remains a challenge. Between 2001 and 2006, the three main clinical questionnaires currently used for the diagnosis of neuropathic pain (6) were developed. These questionnaires are the “Leeds assessment of neuropathic symptoms and signs” pain scale (LANSS) (7), the “douleur neuropathique en 4 questions” questionnaire (DN4) (8), and the painDETECT questionnaire (9). At the time of their publication (2001–2006), the objective of these questionnaires was clearly to distinguish neuropathic pain from nociceptive (non-neuropathic) pain, and the concept of nociplastic pain was not yet introduced and adopted. This history of the progression of knowledge, concepts and definitions of the mechanisms of chronic pain actually constitutes an intrinsic and underestimated limitation to the use of these questionnaires in current clinical practice.

The development and validation of the LANSS, DN4 and painDETECT questionnaires were based on the same design, consisting of the characterization of sensory descriptors whose prevalence differed between two populations of patients with neuropathic pain vs. nociceptive (non-neuropathic) pain. For the three questionnaires, the validation studies included fairly similar clinical conditions both in the neuropathic pain group (peripheral nerve trauma, painful polyneuropathy and postherpetic neuralgia), and in the nociceptive (non-neuropathic) group (osteoarthritis, inflammatory arthropathies and low back pain) (Table 1). However, the most interesting point is to consider the excluded clinical conditions. Fibromyalgia, a typically nociplastic condition, was an exclusion criterion for the DN4 questionnaire study and not mentioned for the validation of the other two questionnaires, but in fact, no patients with fibromyalgia were included in either of these studies. Thus, the three questionnaires were developed without considering nociplastic pain as a condition of exclusion from neuropathic pain, which can be intrinsically explained by the fact that the concept of nociplastic pain is subsequent to the studies that validated these questionnaires.

**Table 1:** Clinical conditions included and excluded in the neuropathic and nociceptive (non-neuropathic) group populations in the LANSS, DN4, and painDETECT studies.

LANSS (Ref. 7)	DN4 (Ref. 8)	PainDETECT (Ref. 9)
Neuropathic pain (*n* = 30)	Neuropathic pain (*n* = 89)	Pain of predominantly neuropathic origin (*n* = 228)
Post-surgical neuropathy (*n* = 8)	Nerve trauma (*n* = 44)	Post-herpetic neuralgia
Post-traumatic neuropathy (*n* = 6)	Postherpetic neuralgia (*n* = 12)	Painful polyneuropathy
Lumbar radiculopathy (*n* = 5)	Painful polyneuropathy (*n* = 12)	Nerve trauma
Complex regional pain syndrome I (*n* = 3)	Benign tumor (*n* = 1)	Neuropathic low back pain
Cervical radiculopathy (*n* = 2)	Spinal cord injury (*n* = 5)	
Peripheral neuropathy (*n* = 2)	Post-stroke pain (*n* = 11)	
Post-herpetic neuralgia (*n* = 2)	Multiple sclerosis (*n* = 4)	
Phantom limb pain (*n* = 1)		
Lumbosacral malignant plexopathy (*n* = 1)		
Nociceptive pain (*n* = 30)	Non-neuropathic pain (*n* = 71)	Pain of predominantly nociceptive origin (*n* = 164)
Low back pain (*n* = 12)	Osteoarthritis (*n* = 40)	Visceral pain
Arthropathies (*n* = 6)	Inflammatory arthropathies (*n* = 23)	Osteoarthritis
Visceral pain (*n* = 2)	Mechanical low back pain (*n* = 8)	Inflammatory arthropathies
Cervical spine pain (*n* = 1)		Mechanical low back pain
Peripheral vascular pain (*n* = 1)		
Repetitive strain injury (*n* = 1)		
Malignant visceral pain (*n* = 4)		
Malignant chest wall pain (*n* = 2)		
Bone metastases (*n* = 1)		
Exclusion criteria	Exclusion criteria	Exclusion criteria
Not detailed	Fibromyalgia	Cancer pain (malignancy)
	Lumbar or cervical radiculopathies	Compression fracture
	Cancer pain	Ankylosing spondylitis
	CRPS type I	
	Headache	
	Visceral pain	

Data issued from Ref. 7: Bennett (2001) (7), The LANSS Pain Scale: the Leeds assessment of neuropathic symptoms and signs. Ref. 8: Bouhassira et al. (2005) (8), Comparison of pain syndromes associated with nervous or somatic lesions and development of a new neuropathic pain diagnostic questionnaire (DN4). Ref. 9: Freynhagen et al. (2006) (9), painDETECT: a new screening questionnaire to identify neuropathic components in patients with back pain.

It should also be noted that the characteristics of nociplastic pain go beyond the somatosensory domain and often include the presence of comorbidities, encompassing different cognitive (attentional) and affective (emotional) aspects of chronic pain (10). These additional criteria are not considered by current neuropathic pain questionnaires, further limiting their ability to make a differential diagnosis with nociplastic pain.

Additionally, these questionnaires establish the diagnosis of neuropathic pain on a cumulative score, meaning that the more sensory descriptors the patient presents, the more likely it is that the pain is neuropathic. However, nociplastic pain is characterized by central sensory sensitization (11) that typically results in the patient perceiving a variety of sensory symptoms, including neuropathic-like symptoms. For example, “my pain feels like burns, electric shocks or cramps” and “my pain is accompanied by other unusual sensations throughout my body such as pins and needles, tingling or numbness”, are items of the Fibromyalgia Rapid Screening Tool (FiRST), a validated questionnaire for the diagnosis of fibromyalgia (12). The same sensory descriptors are found in neuropathic pain questionnaires, and therefore, a high score on these questionnaires may well reveal nociplastic rather than neuropathic pain.

Conversely, some typically neuropathic conditions are characterized by very few different sensory descriptors, such as trigeminal neuralgia, which is a recognized cause of neuropathic pain (5), and is associated with symptomatology limited to stabbing or electric shock-like pain attacks (13). This condition is therefore characterized by low scores on the LANSS, DN4, and painDETECT questionnaires, below their cutoff score for the diagnosis of neuropathic pain, but is nevertheless undeniably neuropathic. This inconsistency is explained by the fact that no patients with trigeminal neuralgia were included in the validation studies of these neuropathic pain questionnaires.

Thus, the LANSS, DN4, and painDETECT questionnaires have intrinsic flaws in their development and therefore in their application for the diagnosis of neuropathic pain. The first inaccuracy concerns the patient profile considered in the validation studies, as no nociplastic pain conditions were included for the differential diagnosis, while some neuropathic pain conditions, likely to produce very specific symptoms, such as trigeminal neuralgia, were also not included. Furthermore, the diagnosis of neuropathic pain by these questionnaires is based on a high score, which results from the presence of a variety of different sensory symptoms and therefore favors a central sensitization process rather than specific neuropathic pain. Furthermore, the inclusion of patients in the validation studies was based on an etiological diagnosis, not excluding a secondary central sensitization process. These questionnaires were therefore not developed to differentiate neuropathic pain with secondary central sensitization from nociplastic pain with primary central sensitization, two situations that can present similar clinical sensory descriptors.

Two other questionnaires, the Neuropathic Pain Questionnaire (14) and the IDPain questionnaire (15), have been proposed for the diagnosis of neuropathic pain, but are less emphasized in international guidelines (6). These questionnaires, however, have the same design and limitations as the three questionnaires detailed in this article. They were actually published in the same period (2003 and 2006) as the other questionnaires, before the introduction of the concept of nociplastic pain.

Thus, the LANSS, DN4 and painDETECT questionnaires should be used with caution as screening tools for the diagnosis of neuropathic pain, as they are unable to differentiate neuropathic pain from nociplastic pain. First, because they did not take into account the existence of nociplastic pain in their development (for an obvious historical reason). Second, because high scores that make the diagnosis of neuropathic pain with these tools are more likely to be observed in cases of nociplastic pain or secondary central sensitization process. On the other hand, these questionnaires do not allow for the diagnosis of neuropathic pain in certain neuropathic conditions limited to a single type of sensory descriptors. Thus, these questionnaires could be more useful for monitoring the evolution of a chronic pain syndrome or the efficacy of an intervention aimed at reducing central sensitization. It is necessary to reexamine the use of these questionnaires in clinical practice, and in particular their value in establishing a definite diagnosis of neuropathic pain. There is therefore an implicit justification for developing new standards for the application of these questionnaires and for defining new ones for differential diagnosis.
